# Developing a patient-centered outcome for targeting early childhood obesity across multiple stakeholders

**DOI:** 10.1186/s40608-018-0216-2

**Published:** 2018-12-03

**Authors:** Byron A. Foster, Paula Winkler, Kelsey Weinstein, Deborah Parra-Medina

**Affiliations:** 1 Departments of Pediatrics and Dermatology, Oregon Health & Science University, Portland, OR USA; 2 Center for Research to Advance Community Health, University of Texas Health San Antonio, San Antonio, TX USA; 3 Latino Research Initiative, University of Texas at Austin, Austin, TX USA

**Keywords:** Patient-centered outcomes research, Growth charts, Goals, Shared decision making, Obesity, Preschool children, Latino, Low-income population, Positive deviance, Delphi technique

## Abstract

**Background:**

Patient-centered outcome measures for childhood obesity are limited. Identifying outcomes that patients and families consider important could be a viable avenue for better engagement of patients and interventions that are efficacious and acceptable to patients. Latino children experience high rates of obesity, and under-recognition of obesity in preschool aged children is common.

**Methods:**

We used growth chart data to identify low-income, Latino children 2–5 years of age with obesity who decreased their adiposity (positive deviants) and a set of controls. We used qualitative interview data to identify themes around goals parents used in addressing weight. Then, we applied a modified Delphi approach across groups of caregivers and providers to identify common goals. We conducted focus groups to explore conflicts and congruency between caregivers and providers related to goals. Using the focus group data, we developed a decision tool for use between patients and providers relevant for early childhood obesity.

**Results:**

We identified 257 children who successfully reduced adiposity (positive deviants) from 1621 eligible growth charts. From interviews with 44 parents (21 positive deviants and 23 controls), we coded and categorized outcomes such as increased happiness, clothing size and improved activity. We recruited 81 parents, grandparents and health care providers to participate in the modified Delphi process of ranking outcomes by importance and feasibility. Focus groups (2, total *n* = 24) suggested potential methods for a common framework to discuss goals, including a modified growth chart. We created a decision-tool that incorporated a growth chart and a section for discussion of patient-centered goals. A final focus group (1, *n* = 10) provided feedback on the tool as acceptable and potentially useful.

**Conclusions:**

The development of a patient-centered tool around achieving a healthy weight in early childhood identified common goals between providers and parents. While the tool has been developed, prospective testing of this patient-centered tool and its effects on engagement, parent motivation, and behavior change would be a useful next step.

**Electronic supplementary material:**

The online version of this article (10.1186/s40608-018-0216-2) contains supplementary material, which is available to authorized users.

## Background

The prevalence of obesity in preschool aged children continues to increase, [1] and the evidence suggests that early weight status tracks into adulthood [2, 3]. However, preschool age children experience under-diagnosis of obesity, even by providers [4]. Parents of children in this age group also consistently underestimate their child’s weight status when they have obesity [5, 6]. Latino/a children experience one of the highest rates of obesity of any racial or ethnic group, and Latino/a children from low-income families experience an even greater risk of persistent obesity [7].

One of the challenges in addressing obesity in this age group is the outcome commonly used by providers to assess and track weight status. BMI (Body Mass Index) percentile and z-scores are not well understood by most parents [8]. Growth charts are even less likely to be used in language-incongruent encounters, a significant barrier for many Latino/a families [9]. In lieu of more or different education on anthropometric measures which some have argued are associated with perpetuating stigma, [10] examining outcomes that are patient-centered may be a viable alternative.

Patient-centered outcomes may improve clinical care and assist in designing interventions that are meaningful to patients. Integration of patient-centered outcomes has been shown to improve care in several chronic conditions including diabetes and rheumatoid arthritis [11]. We are aware of only one study that examined patient-centered outcomes related to obesity management in children [12]. That study identified body image, bullying, and athletic ability as outcomes that school-aged children identified as important to them [12]. A study in adult bariatric surgery examined patient-centered care to assist with decision making and better define patient-specific expectations for weight loss post-operatively [13]. In this study, we attempted to address this gap by identifying and defining potential patient-centered outcomes associated with weight status in preschool age children from low-income, Latino/a families. We used a positive deviance approach to identify families of children who were successful in addressing their weight status and learn from them about the outcomes they used while addressing their weight. We used a combination of qualitative data to generate potential outcomes, a modified Delphi method to develop consensus across both patients and providers, and finally, focus groups to guide the development of a tool that providers and families could jointly use to address weight status in this population.

## Methods

**Fig. 1:**
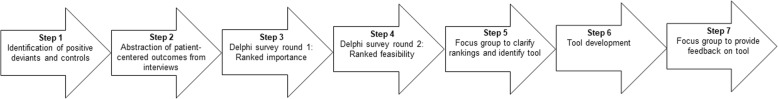
Process of the study steps outlining data collection, interviews, multiple surveys and focus groups leading to development of patient-centered outcome tool

Overall, this project used a mixed-methods approach, employing principles outlined by the Patient Centered Outcomes Research Initiative (PCORI) Methodology Committee, [14] including a broad definition of stakeholders, using qualitative data to identify outcomes relevant to those stakeholders, and using methodologically sound techniques to identify consensus. We used qualitative data from semi-structured interviews with parents to identify potential patient-centered outcomes for obesity, and then engaged a broader range of stakeholders to derive consensus on outcomes that were both important and feasible using a modified Delphi technique (Fig. 1). We then utilized focus groups composed of parents and physicians to assist in identifying an outcome meaningful to both groups. Using this information, we developed an outcome assessment tool. Finally, we used a subsequent independent focus group of parents to gather feedback on the outcome assessment tool generated from this process.

### Identification of positive deviants and controls (step 1)

We used the concept of positive deviance to categorize and identify families for this study. Positive deviance is the idea that among those at highest risk for a poor health outcome, there are some who deviate in a positive way, or succeed despite adversity [15]. In applying this concept to the problem of obesity, we defined the high risk group for continued obesity as children who had been obese in early childhood who were publicly insured and Latino/a, described in detail previously [16]. The rationale for using positive deviants to generate patient-centered outcomes was that they were successful in achieving a reduction in adiposity where so many fail. Our theory was that positive deviants, or more specifically their parents, focused on a different outcome to measure their progress compared with controls.

We used growth chart data of children aged 2–5 years and applied a latent class linear mixed model with a linear link function to identify children with a negative growth trajectory over time who started out at an obese weight (>95th percentile for age and sex). Growth chart data were extracted from a large health system with both academic and community providers that serves about one third of the children in San Antonio, Texas, the 7th largest city in the United States. Growth trajectories were categorized using BMI z-scores with CDC (Centers for Disease Control and Prevention) cut-offs for obesity [17]. Children with a negative growth trajectory were categorized as potential positive deviants. Children whose growth trajectory was stable or increasing were categorized as controls. Children whose parents reported Latino/a ethnicity and public insurance were included. Parents were recruited via phone to participate in a semi-structured interview in their home or at a community site with as many positive deviant families as possible recruited and a comparable number of controls. We completed 44 interviews, each lasting about an hour.

### Abstraction of patient-centered outcomes from interviews (step 2)

The data from each semi-structured interview conducted with both positive deviants and control patients were analyzed specifically for parental motivations and potential outcomes related to their child’s weight status. While other aspects of the interview are more open-ended as described previously, [16] this portion was relatively focused. Some of the specific questions that elicited responses along these lines were: Do you think your child is at a healthy weight? Why or why not? Who specifically in the family made goals regarding your child’s weight? What were those goals? How did you come up with them? How did you measure success for your child as you tried to achieve these goals? How did you keep track and know that things were headed in the right direction? The interviews were transcribed in the language of the interviewee (English or Spanish) and then professionally translated and checked by a native Spanish speaker. We used the theoretical approach of qualitative description [18, 19] to the coding of this section of the interview, given the relatively constrained nature of the data. Interviews were coded by at least two independent coders. Coders initially identified outcomes overall as a code using a deductive approach of what an outcome would be within the interview, and then re-coded these outcomes into sub-codes independently. Discrepancies in these sub-codes applied were resolved by a mix of discussion and involvement of a third coder, as necessary. We used Dedoose software to facilitate the coding process (Los Angeles, CA).

### Delphi survey round 1 (step 3)

We identified each of the outcomes that parents described in the semi-structured interview as something they used to guide their efforts around their child’s weight. We then used a modified Delphi method in the following manner [20]. We recruited eight main groups to participate in two rounds of the Delphi survey process and to discuss appropriate outcomes. The groups were parents (mothers and fathers), grandparents (grandmothers and grandfathers), early childcare educators, doctors, nursing staff and community health workers. Parents and grandparents were recruited from community-based settings by a community health worker; grandparents who were the primary caregivers for a child were excluded from the study. Grandparents were only asked if they had ‘regular interactions’ with a grandchild to be included. Inclusion criteria for the caregivers (parents and grandparents) were Latino/a ethnicity and having a 2–5 year old child or grandchild; caregivers were also compensated for their time with a $10 gift card to a local grocer. Early childcare educators were recruited from a local Head Start program that serves 2–5 year old children. Primary care doctors (both pediatricians and family medicine doctors) were recruited from academic and community settings to participate and could be of any ethnic background, age or sex. The survey process could be completed in either English or Spanish. Survey items were piloted with a separate, small sample of parents and providers prior to implementation to refine verbiage and ensure congruence between the items’ intent and comprehension. A community health worker administered the survey to caregivers and was present for any further clarification or explanation for both rounds.

In the first round, we asked which outcome would be the most important for a family of a 2–5 year old child to use in trying to achieve a healthy weight. A scale of 1–9 was used with 1 being the most important, 9 being the least.

### Delphi survey round 2 (step 4)

In the second round, we took the results from the first round survey and presented them back to the same groups of participants. We asked participants to assess the feasibility of using each of the outcomes in families: “We would also like your opinion on how easy or hard it would be for families of 2-5 year old children to use the outcomes listed.” We included only outcomes ranked in the first round as being in the top three for any sub-group of participants (doctors, mothers etc.).

### Focus group to clarify rankings and identify tool (step 5)

We recruited participants from each sub-group of survey participants to participate in focus groups around the findings to reconcile the conflicting rankings between groups and enrich the understanding of the rankings. We held two focus groups – one for English speakers and one for Spanish speakers. A psychology PhD student with a background in focus groups facilitated both groups, using engagement activities and free word association to open discussion with more specific questions following. Specific questions dealt with the outcomes identified through the surveys, probing for possible explanations for discrepancies between rankings by groups. We also asked about potential ways in which a common language could be developed for discussing obesity and outcomes related to obesity in early childhood. The focus groups were recorded, translated, and professionally transcribed. The data were analyzed using a mixed deductive-inductive approach. Codes were applied by two independent coders, using Dedoose software to facilitate the coding process (Los Angeles, CA).

### Tool development (step 6)

The modified Delphi process and the subsequent focus groups identified outcomes and potential areas to improve communication around weight status in preschool aged Latino/a children. We then examined the tools in current use by physicians and other health professionals related to weight. The Delphi process along with prior work highlighted that the tools used for BMI percentile plotting could be improved [8, 9, 21]. The tool development step intentionally merged the BMI percentile plot used by physicians with the patient-centered outcomes identified, with input on that process provided by a Translational Advisory Board composed of public health representatives and other community leaders.

### Focus group to provide feedback on tool (step 7)

We recruited a group of parents of 2–5 year old children who had not participated at all in the Delphi survey process to a separate focus group designed to provide feedback on the practical utility of the tool we aimed to develop related to obesity. We used hypothetical data to plot children and asked for feedback on the tool, including its interpretation and the use of the goals. As above, the focus group discussion was transcribed, and codes were applied by two independent coders, using Dedoose software to facilitate the coding process (Los Angeles, CA). A deductive approach examining comments related to utility of the tool was applied.

## Results

### Identification of positive deviants and controls (step 1)

From the chart review, 1621 children were identified as potentially eligible by virtue of being Latino/a, obese and 2–5 years of age. Of these, 257 were identified as potential positive deviants by virtue of their decreasing weight trajectory; the rest had either a stable or increasing weight trajectory and were classified as controls. We recruited and completed the semi-structured interview with 21 positive deviants and 23 controls. All participants were Latino/a, 24 (55%) preferred English, 22 (50%) reported an annual income of <$25,000, and 31 (70%) had a high school level education or lower [16].

### Abstraction of patient-centered outcomes from interviews (step 2)

The analysis of positive deviants’ semi-structured interviews compared with controls singled out distinct goals and methods of measuring success (Table 1). Parents in the positive deviant group described multiple ways they measured success related to their child’s habits or weight that included: improved body shape, stable or improving clothing size for their age, actual weight using a scale, ability to play and run, increased happiness, having more energy, increased activity, and bringing their child to a doctor’s appointment for assessment. Some control parents also discussed goals including body shape and bringing the child to a doctor. However, the majority of controls in this sample did not identify a problem with their child’s habits or weight and so had not started the process of identifying goals.

**Table 1:** Illustrative quotes from positive deviants related to parent-described goals

Body shape: You could just see it with him. I was making him a bath. His muscles are coming up.	
Body weight: Since we have scale in my bedroom and it’s available at any time, before we get in the shower we weigh ourselves.	
Clothing size: I would try to, at least, get her down a size or 2 in her clothes. She’s been maintaining for about a year already because she’s in 7/8 s right now. A year ago she was in this size and they were fitting her snug so I’m glad about that.	
Improved activity: Before, he was real slow. Now, he’s starting to get more active and a little bit more faster and a little bit more encouraged to do what the other kids are doing.	
Decreased bullying: They used to make fun of him, criticize him because he couldn’t run. He didn’t like it. I try to prevent it now because I’m like, That’s an ugly feeling. “I wouldn’t want my kids to go through that again.”	
Happiness: “I think being happy and having structure”	

### Delphi survey round 1 (step 3)

There were 81 participants (42% health professionals) who completed the first stage of the Delphi. Of those who completed the first stage, 100% of caregivers reported Latino/a ethnicity and 50% (*n* = 9) of providers reported Latino/a ethnicity. Of all participants, 40% (*n* = 49) completed the survey in Spanish, and the mean age was 45 years (SD = 13), range 22–78 years (Additional file 1: Table S1).

Overall, the three highest-ranked items across groups were increased happiness, decreasing BMI percentile and improved activity (Table 2). The three lowest-ranked items were more energy, improved body shape and better ability to play. There were some notable differences in the relative ranking of outcomes across the different groups, with fathers ranking decreased bullying or harassment in their top three while no other group did so (Table 2). Neither mothers nor fathers ranked decreasing BMI percentile highly whereas each professional group (nurses, doctors, community healthcare workers) ranked that outcome highly.

**Table 2:** Outcomes from the first Delphi round—ranking of *importance*, reported as mode (median) rank within each participant group

	Overall (*n* = 81)	Mother (*n* = 11)	Father (*n* = 11)	Grandfather (*n* = 10)	Grandmother (*n* = 10)	Doctor (*n* = 18)	Nurse (*n* = 8)	Educator (*n* = 5)	CHW (*n* = 8)
Improved activity	1 (3)	1 (1)	1 (3)	1 (3.5)	1 (1.5)	4 (3)	3 (4)	3 (3)	1 (2)
Increased happiness	1 (4)	2 (3)	1 (4)	6 (5)	1 (4.5)	1 (4)	1 (2.5)	3 (3)	5 (5)
Decreased BMI percentile	1 (4)	3 (4)	3 (4)	2 (4)	2 (6)	1 (4.5)	2 (2)	1 (1)	3 (4)
Decreased body weight	2 (4)	2 (3)	3 (4)	4 (4)	3 (4)	2 (4)	6 (4.5)	2 (2)	2 (3.5)
Decreased bullying or harassment	4 (5)	8 (5.5)	2 (3)	8 (6)	4 (5)	5 (5)	6 (6)	5 (5)	7 (6.5)
Stable or improved clothing size	4 (6)	4 (7)	9 (7)	3 (4)	3 (4.5)	8 (6)	8 (8)	4 (4)	3 (5)
Better ability to play	5 (5)	5 (6)	9 (5)	8 (5.5)	2 (5)	5 (4.5)	4 (4)	7 (5)	3 (4)
Improved body shape	8 (7)	6 (6.5)	6 (7)	3 (6)	7 (7)	9 (8)	7 (7)	6 (6)	9 (7.5)
More energy	9 (6)	2 (4)	4 (6)	9 (9)	6 (6)	2 (4)	2 (3.5)	6 (6)	2 (5)

*CHW* Community Health Worker, *BMI* Body Mass Index

### Delphi survey round 2 (step 4)

Forty-six participants (39% health professionals) completed the second stage of the Delphi. Of the providers who completed the second stage, 55% (*n* = 6) reported Latino/a ethnicity. Of all participants, 46% (*n* = 21) completed the survey in Spanish, and the mean age was 48 years (SD = 14), range 23–78 years. There were no significant differences between the two stages in demographic characteristics.

There were five outcomes for participants to rank for feasibility based on selection by importance in the first round. The two most feasible outcomes across groups were increased happiness and improved activity. Improved activity was ranked highly across groups. The least feasible outcomes overall were decreased body weight and decreased bullying or harassment. Every group except for doctors ranked happiness as the easiest to use. Doctors ranked decreased body weight as the easiest (not decreased BMI percentile, which was ranked 4th) (Table 3).

**Table 3:** Outcomes from the second Delphi round—ranking of *feasibility*, reported as mode (median) rank within each participant group

	Overall (*n* = 45)	Mother (*n* = 9)	Father (*n* = 5)	Grandfather (*n* = 6)	Grandmother (*n* = 7)	Doctor (*n* = 10)	CHW (*n* = 8)
Increased happiness	1 (1)	1 (1)	1 (1)	1 (1)	1 (2)	4 (4)	1 (1)
Improved activity	2 (2)	2 (2.5)	2 (2)	2 (2.5)	2 (2)	3 (2.5)	2 (3)
Decreased BMI percentile	3 (3)	3 (3.5)	3 (3)	3 (3)	2 (3)	4 (3)	3 (3.5)
Decreased body weight	4 (4)	4 (4)	4 (4)	4 (4)	4 (4)	2 (2)	4 (4)
Decreased bullying or harassment	4 (5)	1 (3)	5 (5)	5 (5)	5 (5)	5 (4)	1 (3)

*CHW* Community Health Worker, *BMI* Body Mass Index

### Focus group to clarify rankings and identify tool (step 5)

The Spanish group had 14 participants, composed of two fathers, three mothers, two pediatricians, two community health workers, two grandfathers and two grandmothers. The English group had 10 participants, composed of two fathers, two community health workers, two mothers and four grandmothers.

There was no significant resolution of the discrepancy between the doctors’ views and the caregivers’ - parents and grandparents - views in terms of the feasibility of happiness as an outcome. Caregivers persistently argued that happiness was the more important outcome and that they could easily assess this. Physicians participating suggested that making changes in their diet, for example, might be hard but would be beneficial for their health. The caregivers acknowledged the tension between making healthy changes and short-term happiness, and they continued to emphasize happiness as most important. The parents in the focus groups expressed a desire for better communication from their providers related to healthy lifestyles or addressing obesity. Participants brought up the idea of checking with the child’s doctor to make sure the child is on track or making progress related to their weight status. While healthcare providers were referenced as a source of reassurance and expertise related to a child’s weight; a lack of practical information from the healthcare provider was commonly cited as a frustration. Dietary outcomes were not identified as a potential outcome as no cohesive theme was identified from the semi-structured interviews; however, despite the role of diet not being queried in the Delphi process, there was significant unstructured discussion around different ideas on dietary changes or recommendations.

### Tool development (step 6)

**Fig. 2:**
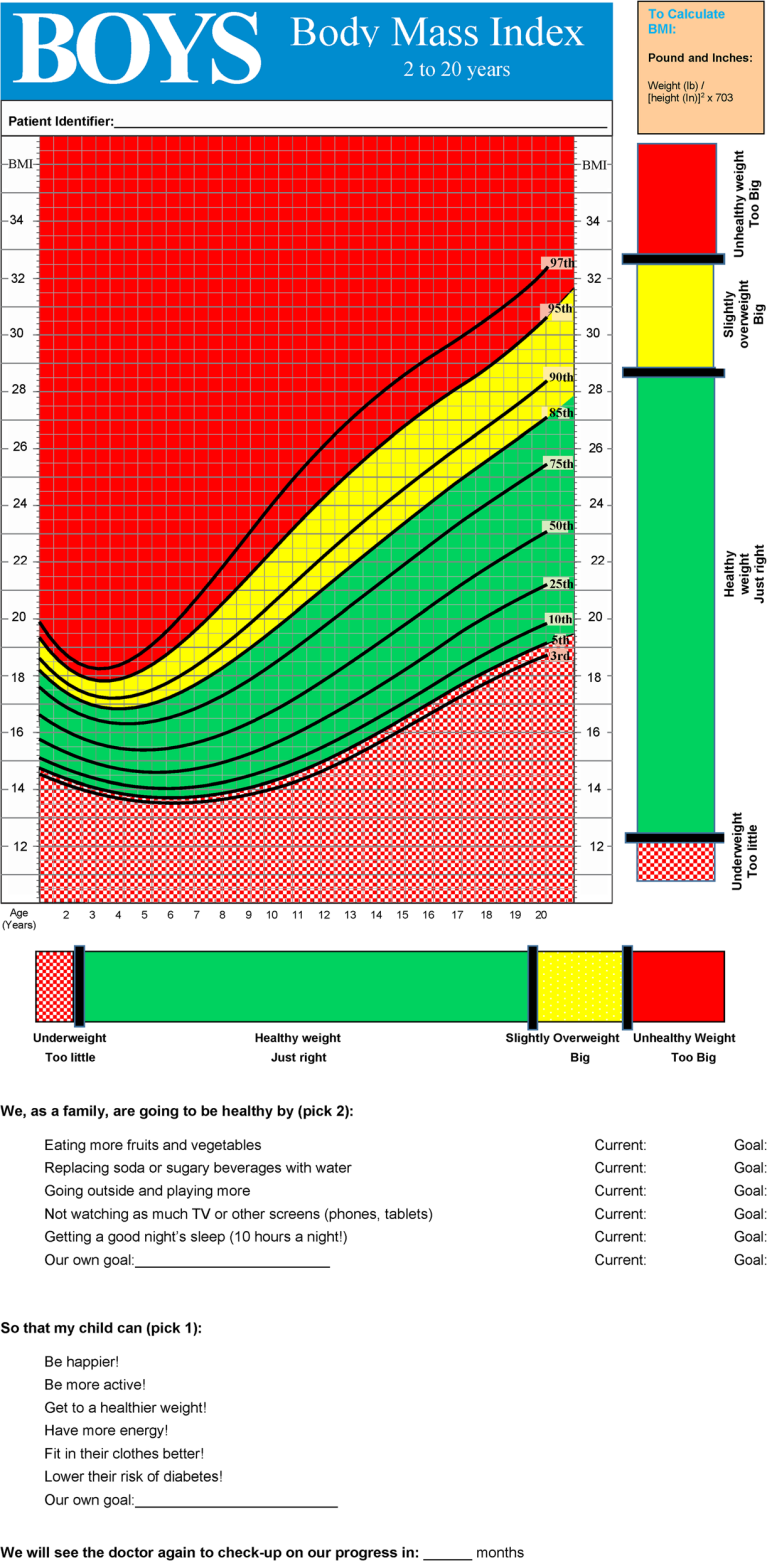
Patient-centered goals and potential behavioral change outcomes used in conjunction with a modified, color-coded ruler and growth chart for facilitating weight status communication (front and back shown in same figure). The CDC growth chart adapted for this figure is not copyrighted (Kuczmarski et al. 2000 [17]), and neither is the ruler adapted for this figure (Cloutier et al. 2013 [21])

The analysis of the qualitative data from positive deviants identified a common theme of validation with their child’s physician. Using these data, we aimed to develop a tool that incorporated evidence-based improvements in the BMI percentile plot and included an operative adaptation of the outcomes identified in the initial steps. This tool incorporated: 1) a ruler to identify on a one-dimensional plane whether the child is at a healthy or unhealthy weight [21], 2) a color-coded growth chart as opposed to the traditional black and white version [22, 23], 3) a set of goals that included the outcomes identified as important and easy to use in the Delphi process, and 4) a set of behavioral changes based on the best available evidence [24, 25] related to reversing an obese weight (Fig. 2). The initial tool design and idea were presented to the Translational Advisory Board with a subsequent iterative process incorporating feedback from the community members comprising the board. The linear ruler was modified to correspond to the same colors as the growth chart with green for healthy weight, yellow for overweight and red for obese weight. The color-coded ruler and growth chart are placed on the same page for ease of transitioning between them, and the sets of goals and potential behavioral changes placed opposite that page to facilitate the conversation. Physicians who were members of the Translational Advisory Board provided informal feedback that they could incorporate the tool into their usual practice.

### Focus group to provide feedback on tool (step 7)

We recruited 10 Latina mothers of 2–5 year old children from a community health center to this focus group. Notably, parents reported seeing a growth chart more often during WIC (Women, Infants, and Children) visits (a federal nutritional supplementation program for low-income families), and less so with their primary care doctor. Most of the mothers liked that the tool facilitated having a conversation related to goals. The perception described was that doctors tell them their child is overweight but do not help them figure out how to address it – and this tool had potential for that. Additionally, the participants in this focus group broadly agreed that some discussion of preventing diabetes should be included in the tool. Otherwise, the additions of color and the ruler were widely perceived as making the growth chart easier to understand.

## Discussion

This paper describes an effort to generate a patient-centered outcome measure for obesity in early childhood, bridging the viewpoints of providers and children’s caregivers. Using a modified Delphi process with a combination of serial surveys and focus groups, we were able to identify some consensus around physical activity as both an important and potentially feasible outcome for both healthcare providers and caregivers. While caregivers recognized the value of the providers’ opinion related to their child’s weight, there were clear concerns over a lack of effective communication using growth charts. We found that a modified growth chart that also incorporated potential patient-centered goals for weight change was more acceptable and easier to understand.

There is one other study we are aware of that examined patient-centered outcomes for childhood obesity [12, 26]. That study, done in Massachusetts, involved families with children identified as positive outliers, using a very similar approach to our classification of positive deviants [12, 26]. The study examined older children (age 6–12) and only a minority were Latino/a; however, they reported very similar outcomes such as clothing size and the ability to be physically active, providing some validation that these outcomes may be a meaningful way that families successfully address weight-related behaviors. The study also found that children described social support as facilitating their success, which was not a finding in our study, probably due to the age difference but we also did not query children directly.

We find it notable that only doctors did not think happiness would be easy to track as an outcome relevant to weight status. This may reflect the fact that doctors have only intermittent interactions with the children, though this may also reflect skepticism of the correlation between happiness and weight status, a viewpoint expressed in the focus groups by physicians. The relative importance of happiness related to weight status has been described previously, with parents asserting that happiness is more important than weight when receiving weight-related feedback in school [27]. Self-esteem and obesity are negatively associated in most studies, though the perception of weight, rather than the child’s weight status itself, may mediate that association [28–30]. There may be a role for using happiness as a goal in order to engage patients, even if the physician cannot measure that as an outcome. There are recent studies linking measures of happiness with physical activity in adults; [31, 32] one of the challenges in measuring happiness in this age group in particular is relying on parental report of child happiness, which tends to correlate more with their own happiness rather than that of their child [33]. Another interpretation may be that happiness is highly important to parents regardless and the responses do not explicitly link weight management behaviors and happiness as an outcome of those behaviors. Given the plausible mechanism between physical activity and happiness, there may be a role for incorporating some discussion of happiness into counseling around weight, though the measurement challenges preclude any immediate steps as an outcome measure for children. In addition, these data do not support any suggestion that children with overweight or obesity are less happy or cannot achieve the same level of happiness as other children – rather they suggest that parents of children highly value happiness and clinicians should consider that in their discussions with families.

The lack of discussion of an outcome related to dietary intake or goals was a notable omission from the qualitative analysis of positive deviants. When this omission was broached in the focus groups, the discussion centered around how eating meals together was central to culture and life among participants. While recognized as an important mediator of a healthy weight, participants did not view diet as a potential outcome.

The focus on the Latino/a population in this study is a potential limitation in applying the findings to other groups, though the remarkable similarity with the findings from the Massachusetts study [26] suggests such findings may be widely applicable. Additional limitations include focusing on publicly-insured subjects and recruitment from one institution. One of the strengths of the study was the inclusion of both English and Spanish speaking participants. The use of the term ‘improved’ to refer to clothing size and body shape by parents in this study did not refer to smaller per se, particularly as children grow with typical development.

We developed the tool (Fig. 2) as a first step towards applying these outcomes in general clinical practice. The tool used prior modifications of the growth chart [21, 23] and incorporated the outcomes derived from the initial steps of this project. This tool should be tested prospectively to examine whether it facilitates greater engagement and understanding with caregivers, particularly amongst non-Latino/a families given the population in which this tool was developed.

## Conclusions

Providers and families ranked outcomes related to child obesity differently initially, though some consensus was reached around physical activity. Patient-centered outcomes related to childhood obesity may provide a means of mitigating the stigma increasingly associated with obesity. Shifting the perspective of providers from an exclusive focus on the growth chart may also assist in forming partnerships with families and community partners to address obesity.

## Additional file

Additional file 1: **Table S1.** Demographic characteristics of modified Delphi participants. Race/ethnicity, preferred language, and age for the 81 participants. (DOCX 12 kb)

## Abbreviations

BMI: Body Mass Index
CDC: Centers for Disease Control and Prevention
CHW: Community health worker
PCORI: Patient Centered Outcomes Research Initiative
WIC: Women, Infants, and Children
