# Supplementary material for: Developing a patient-centered outcome for targeting early childhood obesity across multiple stakeholders
Source: BMC Obes. 2018 Dec 3;5:39. doi: 10.1186/s40608-018-0216-2 (PMC6276184; doi:10.1186/s40608-018-0216-2)
Supplement: Supplementary file 1 — Table S1. Demographic characteristics of modified Delphi participants. Race/ethnicity, preferred language, and age for the 81 participants. (DOCX 12 kb) [file 40608_2018_216_MOESM1_ESM.docx]

**Table S1:** Demographic characteristics of modified Delphi participants

|  | Overall (n=81) | Mother (n=11) | Father (n=11) | Grandfather (n=10) | Grandmother (n=10) | Doctor (n=18) | Nurse (n=8) | Educator (n=5) | CHW (n=8) |
| --- | --- | --- | --- | --- | --- | --- | --- | --- | --- |
| Race/ethnicity  (% Latino) | 68  (84%) | 11 (100%) | 11 (100%) | 10  (100%) | 10  (100%) | 9  (50%) | 4  (50%) | 5  (100%) | 8 (100%) |
| Preferred language (% English) | 49  (61%) | 4  (36%) | 3  (27%) | 4  (40%) | 6  (60%) | 18 (100%) | 7  (88%) | 4  (80%) | 3  (38%) |
| Age, mean in years (SD) | 45.0 (12.7) | 34.1  (8.0) | 38.6  (9.6) | 64.5  (7.6) | 53.7  (7.9) | 39.5  (9.3) | 45.0 (8.4) | 33.6  (8.4) | 53.3 (6.0) |
